# The Merkel Cell Polyomavirus T Antigens Function as Tumor Promoters in Murine Skin

**DOI:** 10.3390/cancers13020222

**Published:** 2021-01-09

**Authors:** Megan E. Spurgeon, Amy Liem, Darya Buehler, Jingwei Cheng, James A. DeCaprio, Paul F. Lambert

**Affiliations:** 1 McArdle Laboratory for Cancer Research, Department of Oncology, University of Wisconsin School of Medicine and Public Health, Madison, WI 53705, USA; aliem@wisc.edu; 2 Department of Pathology and Laboratory Medicine, University of Wisconsin School of Medicine and Public Health, Madison, WI 53705, USA; buehler2@wisc.edu; 3 Department of Molecular, Cellular and Biomedical Sciences, University of New Hampshire, Durham, NH 03824, USA; Jingwei.Cheng@unh.edu; 4 Department of Medical Oncology, Dana-Farber Cancer Institute, Boston, MA 02215, USA; james_decaprio@dfci.harvard.edu; 5 Department of Medicine, Brigham and Women’s Hospital, Harvard Medical School, Boston, MA 02215, USA

**Keywords:** Merkel cell polyomavirus, Merkel cell carcinoma, skin carcinogenesis, T antigens, human polyomaviruses, DNA tumor viruses, viral oncoproteins

## Abstract

**Simple Summary:**

Merkel cell polyomavirus, a recently discovered human virus, is linked to the development of a rare form of skin cancer called Merkel cell carcinoma. The virus does not replicate in cancer cells, yet there is continued expression of viral proteins known as T antigens. The T antigens are believed to contribute to Merkel cell carcinoma development, yet how they do so remains an active area of research. In this study, we used transgenic mice expressing the viral T antigens in their skin to determine at which stage of skin cancer development these viral proteins function. We discovered that the Merkel cell polyomavirus T antigens function as tumor promoters, rather than tumor initiators, in the skin. These findings suggest that other tumor-initiating events may cooperate with the tumor-promoting activities of the viral T antigens, thus providing important insight into how Merkel cell polyomavirus can cause cancer in human skin.

**Abstract:**

Merkel cell polyomavirus (MCPyV) causes the majority of human Merkel cell carcinomas (MCC), a rare but highly aggressive form of skin cancer. We recently reported that constitutive expression of MCC tumor-derived MCPyV tumor (T) antigens in the skin of transgenic mice leads to hyperplasia, increased proliferation, and spontaneous epithelial tumor development. We sought to evaluate how the MCPyV T antigens contribute to tumor formation in vivo using a classical, multi-stage model for squamous cell carcinoma development. In this model, two chemical carcinogens, DMBA and TPA, contribute to two distinct phases of carcinogenesis—initiation and promotion, respectively—that are required for tumors to develop. By treating the MCPyV transgenic mice with each chemical carcinogen, we determined how the viral oncogenes contributed to carcinogenesis. We observed that the MCPyV T antigens synergized with the tumor initiator DMBA, but not with the tumor promoter TPA, cause tumors. Therefore, the MCPyV tumor antigens function primarily as tumor promoters, similar to that seen with human papillomavirus (HPV) oncoproteins. These studies provide insight into the role of MCPyV T antigen expression in tumor formation in vivo and contribute to our understanding of how MCPyV may function as a human DNA tumor virus.

## 1. Introduction

Viruses are the etiological agents of at least 15% of human cancers worldwide [1]. Several viruses with a double-stranded DNA genome, including adenoviruses, papillomaviruses, herpesviruses, and polyomaviruses, possess oncogenic activities in a variety of in vitro and in vivo settings [2]. These DNA tumor viruses have significantly contributed to our understanding of viral oncogenesis and have facilitated many advances in molecular biology and cancer research (reviewed in [2,3]). Given the strong causal relationship of high-risk human papillomaviruses (HPVs) and gamma herpesviruses with human cancers [4,5], these viruses have largely dominated much of the DNA tumor virus research landscape in recent decades. However, the recent discoveries of multiple human polyomaviruses, including one that causes a human skin cancer, have reinvigorated interest in the study of this family of DNA tumor viruses [6].

Merkel cell carcinoma (MCC) was first described in 1972 [7]. Although relatively rare, MCC is one of the most aggressive skin cancers, with a high mortality rate [8]. MCC incidence is predicted to rise dramatically in the coming years due to an increase in the aging population [9]. In addition to advanced age, there are several known risk factors for MCC development, including light-colored skin, ultraviolet (UV) light exposure, and immunosuppression [10,11]. The association with immunosuppression prompted researchers to question whether MCC has a viral etiology. Merkel cell polyomavirus (MCPyV or MCV) was discovered in 2008 through digital transcriptome subtraction, which identified unique, non-human sequences present in human MCC tissues that were subsequently determined to be those of a previously unidentified polyomavirus [12]. MCPyV infection is ubiquitous, largely asymptomatic, and occurs during early childhood [13,14,15,16,17,18,19,20]. While the exact cell targeted for infection remains undetermined, MCPyV most likely exhibits cutaneous tropism for a cell type residing in the skin. MCPyV can be detected in skin swabs of healthy individuals [21] and there is current in vitro evidence that MCPyV can infect and/or replicate in keratinocytes and dermal fibroblasts [22,23,24,25,26].

In at least 80% of MCCs, the MCPyV genome is clonally integrated into the genomic DNA of tumor cells with an integration pattern that frequently preserves the early region of the viral genome [12]. This integration pattern facilitates continued expression of the MCPyV early viral proteins known as tumor (T) antigens, and more specifically the small tumor (ST) and large tumor (LT) antigens [12], both of which have been implicated in transformation/tumorigenesis using in vitro and in vivo studies [27,28,29,30,31,32,33,34]. Within MCC cells, mutations are consistently found within integrated MCPyV DNA genomes that result in expression of truncated forms of LT protein [30,35]. While these C-terminal LT truncations prevent the virus from replicating in the tumor cells, truncated LT proteins retain the LXCXE motif that mediates binding to and inactivation of the cellular tumor suppressor pRb critical in cell cycle regulation [30,36]. ST is also expressed in MCC [37,38] and has oncogenic activity in a variety of assays. MCPyV ST, either alone or in combination with LT, can transform rodent and human fibroblasts in vitro [27,28,29,38] and is tumorigenic in murine skin [31,32,33,34]. Therefore, MCPyV viral genome integration in MCC cells preserves expression of viral proteins with oncogenic activity. To date, MCPyV is the only known polyomavirus to cause cancer in humans.

Several lines of evidence point to MCPyV causing MCC. Integration of the MCPyV genome in MCC cells appears to be an early event in neoplastic progression, as evidenced by clonal integration patterns within individual MCCs and shared integration patterns with distant metastases [12,37,39]. Within MCC tumor cells, viral genome copy numbers generally average at least 1 viral genome copy per cell [39,40,41,42,43], a finding that further supports causality. The importance of the MCPyV ST and truncated LT antigens to MCC oncogenesis is underscored by their retained expression in MCC tumors [37,38,44,45]. Continued expression of the MCPyV T antigens is required for MCC survival and optimal cell growth and proliferation [27,41,46], and the truncated LT and its ability to bind pRb appears to be particularly important in this regard [27,36,47,48]. Recent reports suggest that MCPyV-truncated LT antigen helps drive transdifferentiation of presumably MCPyV-infected MCC precursor cells, at least in part through an ability to increase expression of the Merkel cell specification factor atonal homolog 1 (ATOH1) [49,50]. Expression of survivin (BIRC5), an anti-apoptotic gene, is increased by LT in vitro [51] and is also elevated in the skin of transgenic mice expressing the MCPyV T antigens [32]. In transgenic mouse models, combined expression of MCPyV ST and ATOH1 in squamous epithelial cells induces intraepidermal MCC-like lesions [33]. In our transgenic mouse model, epithelial expression of the MCPyV ST and truncated LT antigens induces hyperplasia, proliferation, and spontaneous tumor development [32]. Furthermore, MCPyV T antigen expression in epithelial cells induces Merkel cell-related gene expression and Merkel cell phenotypes [52]. There are also several other potentially oncogenic functions of the MCPyV T antigens [53]. Overall, the collective evidence that MCPyV contributes to MCC carcinogenesis is ample and continues to grow.

In this study, we sought insight into how MCPyV T antigens cause tumors using a well-validated and widely used multi-stage model of skin carcinogenesis. This model involves the topical application of chemical carcinogens that function as tumor initiators or tumor promoters and thus allow one to define the role of genes or factors in discrete stages of cutaneous tumor development [54,55]. The skin of experimental laboratory animals is treated with a subcarcinogenic dose of a tumor initiator, 7,12-dimethylbenz[a]-anthracene (DMBA), or a tumor promoter, 12-O-tetradecanoylphorbol-13-acetate (TPA), or a combination of both. Skin tumorigenesis requires both tumor initiation and promotion, and malignancy requires progression. If transgenic mice develop tumors after only being treated with TPA (a promoter), this indicates that the transgene product functions as a tumor initiator. Likewise, if tumors develop after only being treated with DMBA (an initiator), this indicates that the transgene product functions as a tumor promoter. Treatment with both DMBA and TPA allows investigation into the role of the transgene in malignant progression. Our laboratory has used this approach, sometimes referred to as a ‘skin painting’ or DMBA/TPA model, to determine the tumorigenic functions of high-risk human papillomavirus oncogenes E5, E6, and E7 [56,57]. In this study, we used MCPyV transgenic mice that express ST and truncated LT antigens in the stratified epithelia and discovered that the MCPyV T antigens can synergize with DMBA, a tumor initiator, to promote tumorigenesis, but do not synergize with TPA, a tumor promoter. Therefore, our results indicate that the MCPyV T antigens function primarily as tumor promoters, and not tumor initiators, in murine skin. These results provide insight into potential mechanisms by which the MCPyV T antigens contribute to MCC neoplastic progression and carcinogenesis.

## 2. Results

### 2.1. Model Validation and Experimental Overview of Studies to Determine the Role of the MCPyV T Antigens in Skin Carcinogenesis

**Figure 1:**
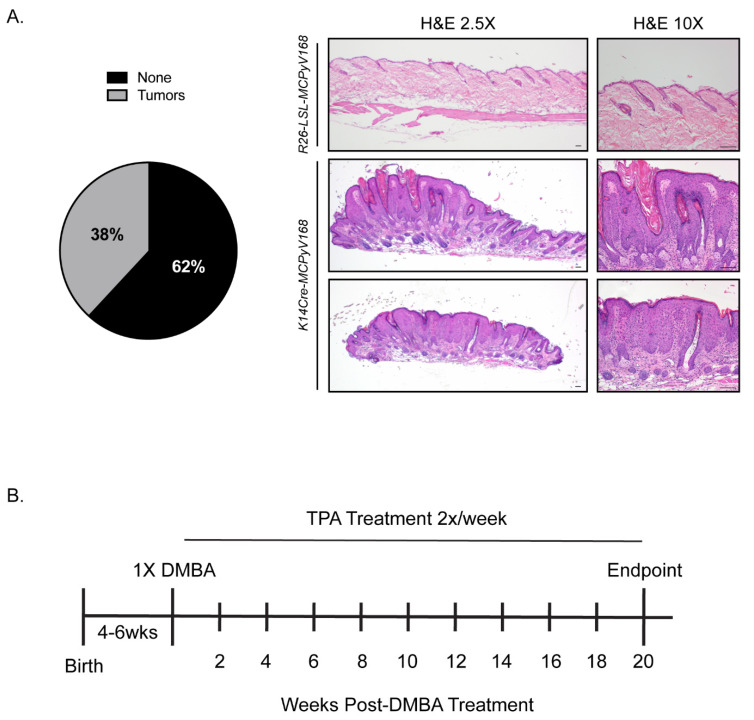
Model validation and experimental overview to determine the role of the MCPyV T antigens in skin carcinogenesis. **(A)** Spontaneous tumor development in untreated *K14Cre-MCPyV168* transgenic mice. The pie chart on the left reflects the percentage of *K14Cre-MCPyV168* mice that did (gray) or did not (black) develop spontaneous tumors. Representative H&E-stained images are shown on the right. Normal skin from *Rosa26-LSL-MCPyV168* mice is shown on top, and sections from two representative spontaneous squamous papillomas that developed on *K14Cre-MCPyV168* mice are shown in the middle and bottom panels. All scale bars = 100 μM. **(B)** Experimental overview of DMBA and TPA treatment regimens in skin carcinogenesis studies. At 4–6 weeks of age, areas of dorsal skin were shaved and prepared in three groups of mice: *Rosa26-LSL-MCPyV168*, *K14E6/E7*, and *K14Cre-MCPyV168*. For mice treated with DMBA only, a one-time topical treatment was applied to the shaved dorsal skin. For mice treated with TPA only, topical treatment was performed twice a week for 20 weeks. For DMBA+TPA treatment, these topical treatments were combined. Mice were monitored for tumor development every 2 weeks during the 20 week treatment period. The average number of tumors per mouse per group was quantified at each time point.

We previously reported that keratin 14 (K14) promoter-driven expression of MCC tumor-derived MCPyV small T and truncated LT antigens in murine skin promotes severe epithelial phenotypes [32]. These *K14Cre-MCPyV168* transgenic mice also spontaneously develop benign epithelial tumors on their skin. Prior to beginning our studies, we sought to verify that our current colony of *K14Cre-MCPyV168* transgenic mice replicate the epithelial tumorigenesis phenotype that we previously observed. Therefore, *K14Cre-MCPyV168* transgenic mice (n = 42) were monitored over the course of 28 weeks and scored for tumor development. Consistent with previous observations, *K14Cre-MCPyV168* mice developed overt phenotypes and approximately 38% (n = 16/42) of mice developed benign epithelial skin tumors at some point during the 28 week period (Figure 1A, left). Consistent with our previous observations, MCPyV T antigen-induced skin tumors contained hyperplastic epithelia and histopathology indicative of non-invasive exophytic lesions with varying degrees of dysplasia (benign papillomas) (Figure 1A, right). While the observed incidence of spontaneous tumor development was slightly lower than our previously reported observations (46% incidence; n = 16/35) [32], it was not significantly different (*p* = 0.64; Fisher’s Exact Test). These findings indicated that the MCPyV T antigens expressed in *K14Cre-MCPyV168* transgenic mice were functioning as expected and allowed us to move forward with our study.

To determine the role of the MCPyV T antigens in skin cancer development, we utilized a multi-stage model of skin carcinogenesis that allows one to determine the role of genes in three stages: initiation, promotion, and progression [54,55]. In this model, murine skin is treated with a subcarcinogenic dose of a tumor initiator, 7,12-dimethylbenz[a]-anthracene (DMBA), or a tumor promoter, 12-O-tetradecanoylphorbol-13-acetate (TPA), or a combination of both. Skin tumorigenesis requires both tumor initiation and promotion, and malignancy requires progression. The ability of a gene or transgene to act as a tumor promoter or initiator in the skin can be elucidated by treating mice with DMBA only or TPA only, respectively. For instance, if transgenic mice develop tumors after only being treated with TPA (a promoter), this indicates that the transgene product functions as a tumor initiator. Likewise, if transgenic mice develop tumors after only being treated with DMBA (an initiator), this indicates that the transgene product functions as a tumor promoter. Transgenic mice treated with both DMBA and TPA allows investigation into the role of the transgene in malignant progression.

In these studies, we included three groups of mice: (1) *Rosa26-LSL-MCPyV168* that do not express Cre recombinase and therefore do not express the MCPyV T antigens as a negative control, (2) *K14E6/E7* HPV16 transgenic mice that have been previously tested using the DMBA/TPA model and therefore serve as a positive control [57], and (3) *K14Cre-MCPyV168* transgenic mice expressing the MCPyV T antigens in K14-positive cells of the stratified epithelia. These mice were separated into three different treatment groups: (1) TPA only, (2) DMBA only, and (3) DMBA+TPA (Table 1). We also included a group of untreated *K14Cre-MCPyV168* mice to monitor the background level of spontaneous tumor development. At 4–6 weeks old, a region of the dorsal skin was shaved to prepare an area for topical treatment. Following the experimental design, we previously used for skin painting studies of HPV16 transgenic mice [56,57], the shaved area of skin of mice in the DMBA-only treatment group was treated topically one time with 0.3 μmol DMBA, and mice in the DMBA+TPA treatment group were treated once with 0.01 μmol DMBA. One week later, mice in the TPA-only and DMBA+TPA treatment groups were treated topically with 15 nmol TPA twice a week for 20 weeks (Figure 1B). All mice were evaluated every 2 weeks for the development of squamous papillomas within the treatment area.

### 2.2. The MCPyV T Antigens Function as Tumor Promoters, Not Initiators, in Murine Skin

**Figure 2:**
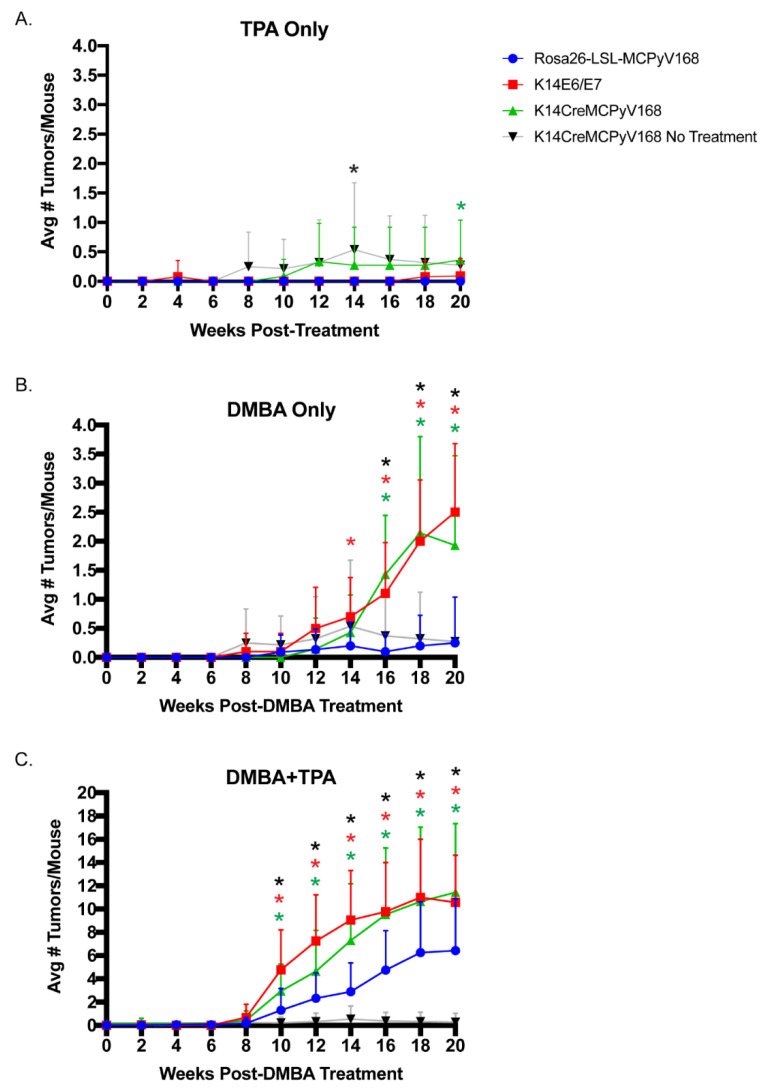
The MCPyV T antigens function as tumor promoters, not initiators, in murine skin and synergize with chemical carcinogens to exacerbate skin tumorigenesis. Groups of *Rosa26-LSL-MCPyV168* (blue data points), *K14E6/E7* (red data points), and *K14Cre-MCPyV168* (green data points) mice were treated topically with (**A**) 15 nmol of TPA twice a week for 20 weeks, (**B**) one time with 0.3 μmol DMBA, or (**C**) treated one time with 0.01 μmol DMBA and then twice a week for 20 weeks with 15 nmol TPA. Tumor incidence over time in an untreated group of *K14Cre-MCPyV168* mice is also included (black data points). At each time point, the average number of tumors/mouse in each group was calculated by dividing the total number of tumors by the total number of mice in each group. Group sizes were adjusted when necessary. A two-sided Wilcoxon rank-sum test was performed on data from each time point to compare the average number of tumors per mouse. Statistical significance is indicated with an asterisk for the comparisons indicated in the figure. Statistical significance indicated in (**A**) black asterisk: *K14Cre-MCPyV168* No Treatment vs. *Rosa26-LSL-MCPyV168 p* = 0.05; green asterisk: *K14Cre-MCPyV168* vs. *Rosa26-LSL-MCPyV16 p* = 0.05; (**B**) black asterisk: *K14Cre-MCPyV168* No Treatment vs. *K14Cre-MCPyV168* Treated *p* < 0.0004; red asterisk: *K14E6/E7* vs. *Rosa26-LSL-MCPyV168 p* < 0.03; green asterisk: *K14Cre-MCPyV168* vs. *Rosa26-LSL-MCPyV168 p* < 2 × 10^−5^; (**C**) black asterisk: *K14Cre-MCPyV168* No Treatment vs. *K14Cre-MCPyV168* Treated *p* < 1.6x10^−9^; red asterisk: *K14E6/E7* vs. *Rosa26-LSL-MCPyV168 p* < 0.008; green asterisk: *K14Cre-MCPyV168* vs. *Rosa26-LSL-MCPyV168 p* < 0.04. The number of mice per group is indicated in Table 1. Error bars indicate standard deviation.

We hypothesized that, if the MCPyV T antigens are able to function as tumor initiators in murine skin, then TPA-treated *K14Cre-MCPyV168* mice will develop significantly more tumors than their untreated counterparts and TPA-treated *Rosa26-LSL-MCPyV168* mice. To determine whether the MCPyV T antigens function as tumor initiators, we treated groups of *Rosa26-LSL-MCPyV168* (n = 17), *K14E6/E7* (n = 13), and *K14Cre-MCPyV168* (n = 12) with TPA only (15 nmol) twice a week for 20 weeks (Figure 1B). We also included a group of untreated *K14Cre-MCPyV168* mice (n = 28) to monitor the incidence of spontaneous tumor development over time. As expected, the negative control *Rosa26-LSL-MCPyV168* mice did not develop any tumors throughout the course of treatment (Figure 2A; blue line). In TPA only treated *K14E6/E7* mice, we observed three total tumors over the course of treatment (1 each at 4, 18, and 20 weeks post-treatment; Figure 2A; red line), a tumor incidence level that was not significantly higher from that observed in the *Rosa26-LSL-MCPyV168* mice (Wilcoxon rank-sum test; all *p*-values >0.2). These findings indicate that the HPV16 E6 and E7 oncogenes do not function as tumor initiators, consistent with our previous studies [57]. In TPA only treated *K14Cre-MCPyV168* mice, we observed a significantly higher tumor incidence compared to *Rosa26-LSL-MCPyV* control mice only at 20 weeks post-treatment (Figure 2A; *p*-value = 0.05). However, this significant increase in tumor incidence likely reflects spontaneous tumor development in MCPyV transgenic mice, as there was no significant difference in tumor incidence between untreated and TPA-treated *K14Cre-MCPyV168* mice at this time point (*p* = 0.43). At no point during the treatment period did the tumor incidence in TPA only treated *K14Cre-MCPyV168* differ significantly from the spontaneous tumor development observed in untreated *K14Cre-MCPyV168* mice (Figure 2A; black line; all *p*-values > 0.13). Together, these data indicate that MCC tumor-derived MCPyV T antigens do not function as tumor initiators in murine skin.

We next tested whether the MCPyV T antigens could function as tumor promoters in murine skin. We hypothesized that, if the MCPyV T antigens are able to function as tumor promoters in murine skin, then DMBA-treated *K14Cre-MCPyV168* mice will develop significantly more tumors than their untreated counterparts and DMBA-treated *Rosa26-LSL-MCPyV168* mice. We previously found that the HPV16 E6 and E7 oncoproteins function as tumor promoters in murine skin [57]. Therefore, tumor incidence in *K14E6/E7* mice should be significantly higher than in DMBA-treated *Rosa26-LSL-MCPyV168* mice and similar to the number of tumors in the DMBA-treated *K14Cre-MCPyV168* transgenic mice, should the MCPyV T antigens function as tumor promoters. Groups of *Rosa26-LSL-MCPyV168* (n = 22), *K14E6/E7* (n = 10), and *K14Cre-MCPyV168* (n = 14) mice were treated with a single dose (0.3 μmol) of the tumor initiator DMBA and monitored for tumor development every 2 weeks for 20 weeks (Figure 1B, Figure 2B). Tumor incidence data from the group of untreated *K14Cre-MCPyV168* mice (n = 28) was included in order to compare the incidence of spontaneous tumor development over time with DMBA-induced tumors. There were no significant differences among any of the groups between 0 and 12 weeks post-DMBA treatment (Figure 2B). At 14 weeks post-treatment, the number of tumors in *K14E6/E7* mice rose to a level significantly higher than *Rosa26-LSL-MCPyV168* mice (*p* = 0.02). Starting at 16 weeks and continuing until the endpoint of 20 weeks post-treatment, the average number of tumors per mouse in DMBA-treated *K14E6/E7* mice (red line) and DMBA-treated *K14Cre-MCPyV168* mice (green line) increased significantly over DMBA-treated *Rosa26-LSL-MCPyV168* mice (blue line) (Figure 2B; *K14E6/E7* and *K14Cre-MCPyV168* versus *Rosa26-LSL-MCPyV168* mice, all *p*-values < 0.0005). The tumor numbers in the DMBA-treated *K14Cre-MCPyV168* mice at 16, 18, and 20 weeks post-infection were all significantly higher than the number of spontaneously arising tumors in untreated *K14Cre-MCPyV168* mice (black line; 16 weeks, *p* = 0.0004; 18 weeks, *p* = 1 × 10^−5^; 20 weeks, *p* = 1.5 × 10^−5^), indicating that the elevated average number of tumors in DMBA-treated MCPyV transgenic mice was a consequence of carcinogen treatment. At no point during the treatment period did the number of tumors differ significantly between DMBA-treated *K14E6/E7* and DMBA-treated *K14Cre-MCPyV168* mice (all *p*-values >0.08). Taken together, these results indicate that the MCPyV T antigens, like the high-risk HPV16 oncoproteins, function as tumor promoters in murine skin.

### 2.3. The MCPyV T Antigens Synergize with Chemical Carcinogens to Exacerbate Skin Tumorigenesis

To determine the relative contribution of viral genes and carcinogens to malignant progression, animals were treated with both tumor-initiating and -promoting chemicals (DMBA and TPA, respectively). This dual treatment will induce tumor formation even in non-transgenic murine skin. Tumor-bearing mice were then held for an additional period of time to monitor for progression of benign tumors to cancer. However, prior to this holding period, we analyzed whether the MCPyV T antigens can synergize with DMBA and TPA to exacerbate tumorigenesis. Groups of *Rosa26-LSL-MCPyV168* (n = 23), *K14E6/E7* (n = 17), and *K14Cre-MCPyV168* (n = 12) were treated once with 0.01 μmol DMBA and then twice a week with 15 nmol TPA for 20 weeks (dual carcinogen-treated, Figure 1B). The number of tumors was recorded every two weeks and the average number of tumors per mouse calculated at each time point (Figure 2C). In all groups of dual carcinogen-treated mice, the number of tumors began to rise at 10 weeks post-treatment. The average number of tumors in dual carcinogen-treated *K14Cre-MCPyV168* mice was significantly higher than in the dual carcinogen-treated *Rosa26-LSL-MCPyV168* mice at all time points between 10 and 20 weeks post-treatment (all *p*-values < 0.04). This significant increase in the average number of tumors compared to dual carcinogen-treated *Rosa26-LSL-MCPyV168* mice was similar in the dual carcinogen-treated *K14E6/E7* mice (all *p*-values < 0.008). However, despite these similarities, the difference between dual carcinogen-treated *Rosa26-LSL-MCPyV168* and dual carcinogen-treated *K14E6/E7* mice was generally more highly significant, especially between 10 and 14 weeks post-treatment, than the difference between dual carcinogen-treated *Rosa26-LSL-MCPyV168* and dual carcinogen-treated *K14Cre-MCPyV168* mice. At 12 weeks post-DMBA treatment, the average number of tumors in dual carcinogen-treated *K14E6/E7* mice was significantly higher than the number in dual carcinogen-treated *K14Cre-MCPyV168* mice (*p* = 0.03), and the difference between these two groups trended towards significance at both 10 weeks (*p* = 0.07) and 12 weeks (0.09) post-DMBA treatment. However, towards the end of the treatment period at 16, 18, and 20 weeks, the average number of tumors present in dual carcinogen-treated *K14E6/E7* and dual carcinogen-treated *K14Cre-MCPyV168* mice was statistically indistinguishable (16 weeks, *p* = 0.51; 18 weeks, *p* = 0.38; 20 weeks, *p* = 0.79). At all time points between 10 and 20 weeks, the number of tumors that developed in dual carcinogen-treated *K14Cre-MCPyV168* mice was significantly higher than in untreated controls (all *p*-values <1.6 × 10^−9^). These results indicate that the MCPyV T antigens synergize with chemical carcinogens to promote tumorigenesis, and do so at a level similar to that of the high-risk HPV16 E6 and E7 oncoproteins.

### 2.4. Assessment of Malignant Progression

**Figure 3:**
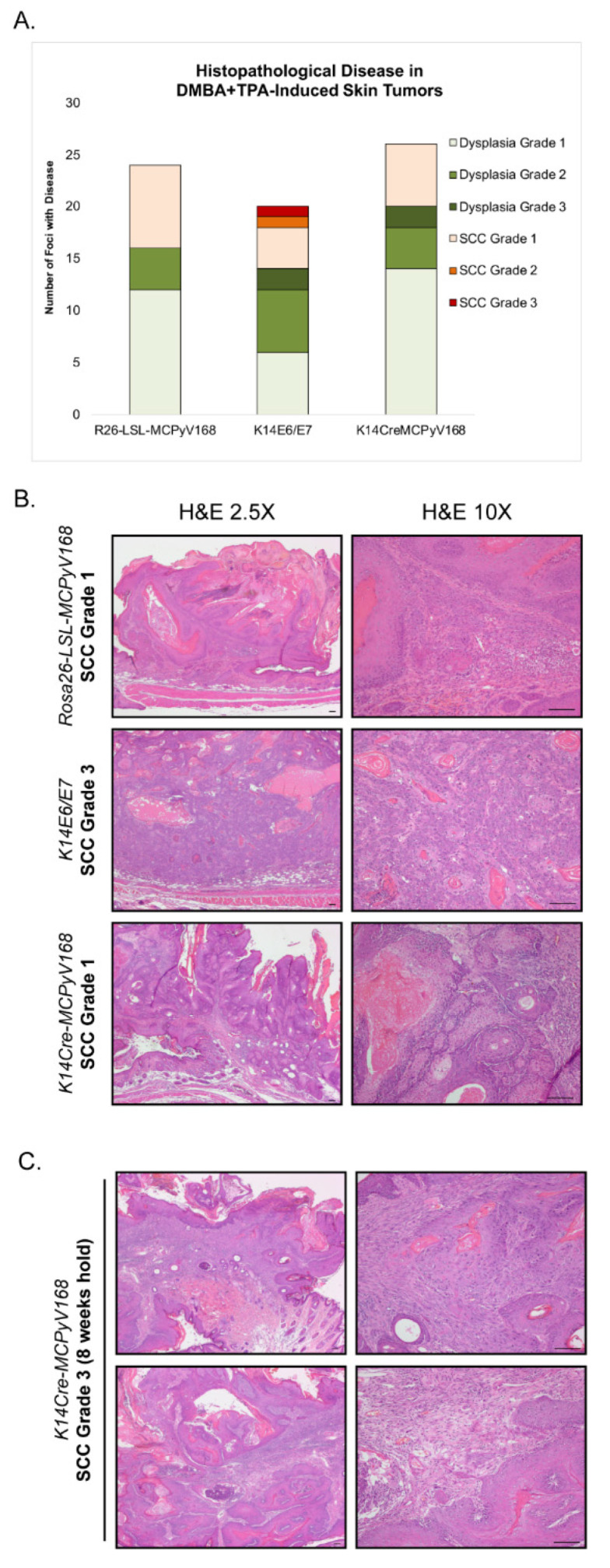
Assessment of malignant progression. (**A**) After the 20 week DMBA+TPA treatment period, tumor-bearing *Rosa26-LSL-MCPyV168*, *K14E6/E*, and *K14Cre-MCPyV168* mice were held without further treatment for an additional 5 weeks. Skin was harvested, sectioned into 5 μM sections and placed on glass slides, and stained with hematoxylin and eosin (H&E). Tissues were evaluated for histopathological disease. Each foci/tumor evaluated was given a score for worst disease among the following grades: Dysplasia Grade 1, Dysplasia Grade 2, Dysplasia Grade 3, Squamous Cell Carcinoma (SCC) Grade 1, SCC Grade 2, or SCC Grade 3. The number of foci with each disease score in each group of mice is indicated in the bar graph. The overall disease severity between groups was not statistically significant (two-sided Wilcoxon rank-sum test; all *p*-values > 0.17 for all comparisons). (**B**) Representative H&E-stained images of tissue sections from tumors harvested from DMBA+TPA-treated *Rosa26-LSL-MCPyV168*, *K14E6/E7*, and *K14Cre-MCPyV168* mice after 5 weeks hold. Images show representative examples of worst disease state present in each treatment group. All scale bars = 100 μM. (**C**) Representative H&E-stained images of tissue sections from tumors harvested from a DMBA+TPA-treated *K14Cre-MCPyV168* mouse after 8 weeks hold. Images show representative examples of worst disease state present. All scale bars = 100 μM.

To evaluate malignant progression, mice in the dual carcinogen (DMBA + TPA)-treated group normally are held for an additional 20 weeks following completion of the TPA treatment to allow time for malignant progression to occur [57]. At the end of this 20 week holding period, tissues were harvested and evaluated for histopathological disease and progression to squamous cell carcinoma (SCC). In this study, several mice developed excessive tumor burden that necessitated humane euthanasia of many of the mice, particularly in the dual carcinogen-treated *K14E6/E7* and dual carcinogen-treated *K14Cre-MCPyV168* groups, well ahead of the 20 week hold period. This compromised our ability to monitor for malignant progression. Nevertheless, we did analyze the histopathology and scored for worst disease in tumors harvested 5 weeks into the holding period from dual carcinogen-treated *Rosa26-LSL-MCPyV168* mice (n = 24 foci from n = 8 mice), dual carcinogen-treated *K14E6/E7* mice (n = 20 foci from n = 6 mice), and dual carcinogen-treated *K14Cre-MCPyV168* mice (n = 26 foci from n = 7 mice). Each tumor/foci was scored as having either No Disease, Squamous Dysplasia Grade 1 (mild), Squamous Dysplasia Grade 2 (moderate), Squamous Dysplasia Grade 3 (severe), Squamous Cell Carcinoma (SCC) Grade 1 (well differentiated), SCC Grade 2 (moderately differentiated), or SCC Grade 3 (poorly differentiated) (Table 2, Figure 3).

In dual carcinogen-treated *Rosa26-LSL-MCPyV168* mice, 67% of tumors (n = 16/24) progressed to precancerous dysplasia (n = 12 Dysplasia Grade 1, n = 4 Dysplasia Grade 2) and the remaining 33% of tumors (n = 8/24) had progressed to SCC Grade 1 within the 5 week holding period (Figure 3A). There was little difference between the disease that developed in dual carcinogen-treated *Rosa26-LSL-MCPyV168* mice and dual carcinogen-treated *K14Cre-MCPyV168* mice. Nearly 77% of tumors evaluated from dual carcinogen-treated *K14Cre-MCPyV168* mice had progressed to dysplasia (n = 14 Dysplasia Grade 1, n = 4 Dysplasia Grade 2, n = 2 Dysplasia Grade 3) and the remaining 23% (n = 6/26) of tumors had progressed to SCC Grade 1 (Figure 3A). There was no statistically significant difference between the overall disease severity in dual carcinogen-treated *Rosa26-LSL-MCPyV168* and dual carcinogen-treated *K14Cre-MCPyV168* mice (*p* = 0.68). In dual carcinogen-treated *K14E6/E7* mice, 70% of tumors progressed to dysplasia (n = 6 Dysplasia Grade 1, n = 6 Dysplasia Grade 2, n = 2 Dysplasia Grade 3). While around the same percentage of tumors from dual carcinogen-treated *K14E6/E7* mice progressed to SCC (30%; n = 6/20) as in the other groups of mice, the SCC grade of severity was slightly elevated such that we identified some tumors that progressed to SCC Grade 1 (n = 4/20), SCC Grade 2 (n = 1/20) and SCC Grade 3 (n = 1/20) (Figure 3A). However, the overall disease severity in dual carcinogen-treated *K14E6/E7* mice was not significantly higher than that in dual carcinogen-treated *Rosa26-LSL-MCPyV168* mice (*p* = 0.33) or dual carcinogen-treated *K14Cre-MCPyV168* mice (*p* = 0.17). Representative histology of worst disease that developed after 5 weeks hold in each group of mice is shown in Figure 3B. We conclude that the MCPyV T antigens do not acutely contribute to significant malignant progression, at least within 5 weeks following DMBA+TPA treatment. We were able to hold one dual carcinogen-treated *K14Cre-MCPyV168* mouse for a total of 8 weeks post-treatment (Figure 3C). Of the 5 total foci we evaluated from this single mouse, 60% (n = 3/5) were dysplastic and 40% (n = 2) developed into invasive SCCs. One cancer was scored as SCC Grade 1 and the other was scored as SCC Grade 3. Therefore, we have reason to believe that, had we been able to hold the mice longer, we might have observed a contribution of MCPyV T antigens to malignant progression.

## 3. Discussion

In this study, we evaluated how the MCPyV T antigens function within discrete stages of tumorigenesis in murine skin using a classical multi-stage model of squamous cell carcinoma development. In this model, both tumor-initiating and -promoting functions are needed for skin tumorigenesis in murine skin and resulting tumors can undergo malignant progression to cancer [55]. We found that MCPyV transgenic mice treated with the tumor promoter TPA failed to give rise to an increased number of tumors relative to the number that spontaneously develop on the skin of *K14Cre-MCPyV168* mice (Figure 1, Figure 2A). Conversely, there was a significant increase in tumor development in MCPyV transgenic mice treated with the tumor initiator DMBA and tumor development occurred at a level similar to that observed in HPV16 transgenic mice (Figure 2B). We also found that the MCPyV T antigens synergized with dual carcinogen treatment to significantly exacerbate skin tumor development, again to a level similar to HPV16 oncoproteins (Figure 2C). Finally, although we were unable to complete our studies on malignant progression as planned, we found some evidence that MCPyV T antigen expression in *K14Cre-MCPyV168* mice could contribute to malignant progression but did not significantly do so within the abbreviated time period of our study (Figure 3). In total, our results demonstrate that MCC tumor-derived MCPyV T antigens function as tumor promoters, and not tumor initiators, in murine skin.

Our finding that MCPyV T antigens function in tumor promotion, but not initiation, raises the question: what factor(s) drive tumor initiation to give rise to MCPyV-positive MCC? We believe there are several possibilities for initiating events during MCPyV-induced MCC pathogenesis. One possibility is MCPyV genome integration, a hypothesis presented in the initial report on MCPyV’s discovery [12]. In a recent study, Starrett and colleagues found that MCPyV genome integration is associated with host genome amplifications and copy number variations (CNVs), similar to what is observed with other integrated oncogenic viruses like HPV [58,59], which the authors speculated could cause genomic instability. Therefore, MCPyV viral genome integration may initiate tumorigenesis by potentiating these potentially mutagenic events. In light of this proposed model of integration-induced tumor initiation by DNA tumor viruses, it is interesting that we also saw no evidence of tumor-initiating roles for the high-risk HPV16 oncogenes E5 [56], E6, or E7 [57] in our transgenic models. Notably, none of our HPV transgenic models nor our MCPyV transgenic model are infection models and do not involve the process of viral genome integration. Our murine models, therefore, effectively obscure any role of viral genome integration in genomic instability and tumor initiation. Nevertheless, viral genome integration is one possible mechanism of tumor initiation during MCC pathogenesis.

Another possibility is that the MCPyV T antigens themselves may contribute to tumor initiation, either through inherent functions or within certain contexts. For instance, the C terminus of the MCPyV full-length LT antigen induces DNA damage and genomic instability during in vitro MCPyV infection [60]. It is unclear when this DNA damage-inducing domain is lost during MCC pathogenesis, although there is evidence that LT truncation occurs before or during integration [61], thus allowing a window of time for LT-induced host genomic instability. ST can induce several chromosomal abnormalities, such as aneuploidy, chromosomal breaks, and micronuclei, in transduced fibroblasts and in ST transgenic mice [62]. Theoretically, ST could also function as a tumor initiator through its association with MYCL and the chromatin remodeling complex EP400, which leads to increased transcription of several pro-oncogenic genes [63]. One such gene is MDM4, which acts with MDM2 to cause p53 ubiquitination and subsequent degradation [64]. There is also evidence that ST expression in the epidermis initiates MCC-like lesions in murine skin, but only when expressed in keratinocytes that are also expressing the Merkel cell specification factor ATOH1 [33]. It is also possible that there is a heretofore unappreciated co-carcinogenic factor that contributes to the initiation events in MCPyV+ MCC. Therefore, the MCPyV T antigens may require cooperation with additional co-factors and/or precise conditions to function as tumor initiators in the complex environment of the skin. This hypothesis seems plausible given the ubiquitous nature of MCPyV infection, yet rare incidence of MCC, in the human population.

It is possible that host factors contribute to tumor initiation during MCC pathogenesis. It is now well established that MCPyV-negative and MCPyV-positive MCC tumor cells contain significantly different mutational landscapes in their host genomes [58,59,65,66,67]. MCPyV-negative MCCs are characterized by an abundance of mutations that bear signatures consistent with UV-mediated mutagenesis, whereas MCPyV-positive MCCs have a low overall mutational burden. While these findings seem to imply that the contribution of somatic mutations to tumor initiation in MCPyV-positive MCC is minor, there may be random or rare mutational events in driver genes whose likelihood to arise over time is increased by the hyperproliferative effects of the MCPyV T antigens. Potential driver mutations have been identified in MCPyV-positive MCCs, with one study finding such mutations in approximately 30% of tumors [67]. For instance, activating gene mutations in the phosphatidyl-3-kinase/AKT/mammalian target of rapamycin (PI3K/AKT/mTOR) pathway are found in MCCs. Activating mutations in *AKT1*, *PIK3CA*, and *HRAS* and loss of function mutations in genes that act as negative regulators of the pathway, such as *PTEN* and *TSC1*, have been detected by multiple groups in MCPyV-positive MCCs [65,67,68,69,70,71]. Along these lines, it is interesting to note that DMBA, the tumor-initiating chemical carcinogen used in this study, is thought to act as a tumor initiator by inducing activating mutations in *HRAS* [72]. We therefore speculate that such DMBA-induced mutations may imitate at least one of the tumor-initiating somatic driver mutations found in MCPyV-induced MCCs, which could then cooperate with the tumor-promoting functions of the MCPyV T antigens to drive tumorigenesis. Interestingly, we have evidence that activating PI3K mutations are sufficient to drive tumorigenesis in HPV16 transgenic mice [73]. In a model of HPV-positive anal cancer, DMBA treatment and the HPV oncogenes serve the tumor initiator and tumor promoter roles, respectively [74]. However, an activating mutation in *PIK3CA* was sufficient to drive anal carcinogenesis in HPV16 transgenic mice without DMBA administration [73]. Therefore, it is reasonable to hypothesize that such driver mutations can also cooperate with the tumor-promoting activity of the MCPyV T antigens in a similar way during MCC pathogenesis.

The multi-stage carcinogenesis model used in this study is an eminent model used to investigate the development of tumors of epithelial origin, most notably squamous cell carcinoma [54,75]. The cellular origin of MCC remains unclear, as does the cell type infected by MCPyV, and both are active areas of research (reviewed in [76]). Despite similarities in gene expression signatures, biomarkers, and histology, Merkel cells are post-mitotic [77] and quite rare within the skin and therefore themselves seem an unlikely precursor to MCC. The current paradigm suggests that MCPyV-positive MCCs derive from an infected precursor cell type that has yet to be identified. Some possibilities include dermal fibroblasts, which can support MCPyV infection and replication in vitro [24], lymphoid pre/pro-B cells [78], and neuronal cells [50]. However, growing evidence supports an epithelial cell of origin. Merkel cells arise from epidermal progenitor cells [77,79] and ectopic expression of the Merkel cell specification factor atonal homolog 1 (ATOH1) in epithelial cells induces Merkel cell development [80]. Interestingly, MCPyV LT expression in keratinocytes has been observed to not only increase ATOH1 expression [50] but also prevent its degradation [52]. Furthermore, combined expression of the MCPyV T antigens and cellular genes GLI1 or ATOH1 induces Merkel cell-like phenotypes in vitro [52] and MCC-like lesions in vivo [33], respectively. A recent study reported sequencing evidence that a MCPyV-positive MCC was derived from an epithelial lineage [81]. While it is possible that the T antigen functions differ in other potential MCC precursor cell types, our evaluation of MCPyV T antigen function in epithelial tumorigenesis determined that these viral proteins function as tumor promoters when their expression is targeted to one likely MCC precursor cell population, K14-positive epithelial cells.

Throughout the course of our studies with *K14Cre-MCPyV168* transgenic mice, we continue to observe considerable similarities between the activities of keratin 14-driven expression of the MCPyV T antigens and the high-risk HPV16 oncoproteins E6 and E7. During our initial characterization of MCPyV transgenic mice, we found that the MCPyV T antigens induced epithelial hyperplasia, cellular proliferation, and E2F-dependent gene expression to the same extent as HPV16 E6 and E7 [32]. Here, we have observed that the MCPyV T antigens and HPV16 oncoproteins act in very similar ways in a model of skin carcinogenesis. The HPV oncoproteins, particularly E5 and E7, function as tumor promoters in murine skin [56,57], while E6 primarily functions in malignant progression [57]. We included *K14E6/E7* transgenic mice in our study, to compare findings to those obtained with the MCPyV transgenic mice, and discovered that the MCPyV T antigens also function as tumor promoters. The tumor incidence in *K14Cre-MCPyV168* mice largely mirrored that in *K14E6/E7* mice following DMBA-only treatment (Figure 2B), and the same was true when both transgenic lines were treated with DMBA+TPA (Figure 2C). These similarities seem to reflect the often overlapping, parallel functions of oncogenic DNA tumor virus proteins [2]. A few observations may suggest that the HPV16 oncoproteins are slightly more potent tumor promoters than the MCPyV T antigens. For instance, tumor incidence in DMBA only treated animals rose to a level significantly higher than the negative control group slightly faster in *K14E6/E7* mice than in *K14Cre-MCPyV168* mice (14 versus 16 weeks, respectively; Figure 2B). While the number of tumors in both groups were significantly higher than in *Rosa26-LSL-MCPyV168* mice, the number of tumors in DMBA+TPA-treated *K14E6/E7* mice were more highly significant than in DMBA+TPA-treated *K14Cre-MCPyV168* mice (Figure 2C). Finally, some of the tumors present in DMBA+TPA-treated *K14E6/E7* mice progressed to a more advanced grade of SCC within the limited 5 week holding period than did tumors in DMBA+TPA-treated *K14Cre-MCPyV168* mice, though the overall severity of disease was not significantly different (Figure 3), which may reflect the actions of E6 in the progression stage [57]. Therefore, while there are functional similarities between epithelial expression of the MCPyV T antigens and HPV16 oncoproteins, there may still be important differences in their underlying mechanisms and/or potency.

There remain several outstanding questions related to the specific role of the MCPyV T antigens in MCC pathogenesis that are raised by our study. For instance, there is still a great deal to learn about the individual and cooperative roles of the truncated LT and ST antigens in MCCs. Both T antigens are expressed in the *K14Cre-MCPyV168* mice used in this study, and it would be interesting to test the role of the individual MCC tumor-derived T antigens using the multi-stage model of skin carcinogenesis. The difficulty we encountered in evaluating malignant progression in our study left us with an unsatisfying level of insight regarding the role of MCPyV T antigens in this stage of carcinogenesis. Because this model is so well studied and utilized, there are several identified areas for optimization related to treatment dose and duration that we can explore in future studies that may increase our ability to study malignant progression [54]. Given its association with MCC [11], we could also adapt this model to study the role of ultraviolet light exposure in MCPyV-associated tumorigenesis and whether it functions more as a mutagen [58,65,67,71] or as an immunosuppressive or immunomodulatory agent [82] in this process. The development of MCPyV transgenic mice provide ample opportunities to further explore the underlying mechanisms of the MCPyV T antigens in MCC pathogenesis and neoplastic progression.

## 4. Materials and Methods

### 4.1. Animals

The MCPyV transgenic mice, named *ROSA26-LSL-MCPyV168*, have been described previously [32]. Briefly, the MCPyV early region, isolated from MCC tumor specimen MCCw168 (MCPyV168; GenBank: KC426954.1), was cloned into vectors containing a LoxP-stop-LoxP cassette (LSL) and the pROSA26PA plasmid. These conditional *ROSA26-LSL-MCPyV168* mice were crossed with transgenic mice expressing Cre recombinase driven by the human keratin 14 (*Krt14* or K14) promoter (*K14Cre*) to generate *K14Cre-MCPyV168* mice. The *K14E6/E7* bitransgenic mice included in this study express the HPV16 E6 and E7 oncogenes driven by the K14 promoter and have been described previously [83,84]. All mice were maintained on the *FVB/N* genetic background. All animal experiments were performed in full compliance with standards outlined in the Guide for the Care and Use of Laboratory Animals by the Laboratory Animal Resources (LAR) as specified by the Animal Welfare Act (AWA) and Office of Laboratory Animal Welfare (OLAW) and approved by the Governing Board of the National Research Council (NRC). Mice were housed at the McArdle Laboratory Animal Care Unit in strict accordance with guidelines approved by the Association for Assessment of Laboratory Animal Care (AALAC), at the University of Wisconsin Medical School. All protocols for animal work were approved by the University of Wisconsin Medical School Institutional Animal Care and Use Committee (IACUC; protocol number M005871).

### 4.2. Genotyping

All transgenic mice used in these studies were verified by PCR genotyping. Genomic DNA was isolated from tail snips and resuspended in water. Separate PCR reactions were used to identify the wild-type or recombined ROSA26 allele, presence of the Cre recombinase gene, and the E6/E7 transgenes. PCR products were evaluated using agarose gel electrophoresis. The following primers were used for genotyping: P1 (5′-AAA GTC GCT CTG AGT TGT TAT-3′), P2 (5′-GCG AAG AGT TTG TCC TCA-3′) and P3 (5′-AGC GGG AGA AAT GGA TAT-3′) specific for the ROSA26 allele; 3069 (5′-TTC CTC AGG AGT GTC TTC GC-3′) and 3070 (5′-GTC CAT GTC CTT CCT GAA GC-3′) for K14Cre; Oligo-2 (5′-GCA TGA CAG CTG GGT TTC TCT ACG-3′) and E6TTL (5′-GCT TAG TTA ACT AAT GCA AAC-3′) for E7, and E7TTL (5′- AGC CTT AGT TAA CTA ACA TTA C-3′) and 709-4 (5′-CCC GGA TCC TAC CTG CAG GAT CAG CCA TG-3′) for E6. All primers were synthesized by Integrated DNA Technologies (Coralville, IA).

### 4.3. Skin Carcinogenesis Studies

At 4 to 6 weeks of age, the dorsal area of mice was shaved to create an area for topical carcinogen treatment and the animals divided into three groups. One group of mice (TPA only) was treated with 12-O-tetradecanoylphorbol-13-acetate (TPA), a promoting agent. The skin of these mice was topically treated with 15 nmol TPA, dissolved in acetone, twice weekly for 20 weeks. Another group of mice (DMBA only) were topically treated once with 0.3 μmol 7,12-dimethylbenz[a]-anthracene (DMBA), an initiating carcinogen, also dissolved in acetone. The third group of mice (DMBA+TPA) was topically treated with 0.01 μmol DMBA and one week later, the same skin region was topically treated with 15 nmol TPA twice weekly for 20 weeks. We also included an additional group of *K14Cre-MCPyV168* mice that were left untreated to serve as a baseline control for the number of tumors these mice spontaneously develop over time [32]. All mice were examined every 2 weeks for tumors, and the average number of gross tumors per mouse was calculated at each time point.

To evaluate malignant progression, mice that had been treated with DMBA+TPA were held following treatment completion and mice were not treated in any way during this time. While the original protocol used in our laboratory to study malignant progression prescribes a 20 week hold, we were only able to hold mice for 5 weeks due to excessive tumor burden in *K14E6/E7* and *K14Cre-MCPyV168* mice. At the end of 5 weeks, skin was harvested, fixed in 4% paraformaldehyde, and embedded in paraffin. All tissue sections were prepared by an experienced histotechnologist by cutting 5 μM serial sections from the paraffin blocks and placing on glass slides. Every 10th section was stained with hematoxylin and eosin (H&E) to facilitate histopathological analysis. At least 20 tumors, or foci, from each group of DMBA+TPA-treated mice held for 5 weeks were selected for histopathological analysis. Each tumor/foci was assessed for squamous dysplasia and keratinizing invasive squamous cell carcinoma according to the standard histopathologic criteria as having No Disease, Squamous Dysplasia Grade 1 (mild), Squamous Dysplasia Grade 2 (moderate), Squamous Dysplasia Grade 3 (severe), Invasive Squamous Cell Carcinoma (SCC) Grade 1 (well differentiated), SCC Grade 2 (moderately differentiated), or SCC Grade 3 (poorly differentiated).

### 4.4. Statistical Analysis

The average number of tumors per mouse was calculated by dividing the total number of tumors present within a given group of animals by the total number of animals present per group at the indicated time point. If animals dropped out of the experiment, either by morbidity due to advanced age or required euthanasia for excessive tumor burden, the group size was adjusted accordingly. Data were compiled and graphs generated using the GraphPad Prism program (Version 8.4.3; last accessed 30 October 2020). A two-sided Wilcoxon rank-sum test was used to compare the average number of tumors per mouse between groups at each time point. To compare disease severity, each histopathological grade was assigned a rank (No Disease = 0, Dysplasia Grade 1 = 1, Dysplasia Grade 2 = 2, Dysplasia Grade 3 = 3, SCC Grade 1 = 4, SCC Grade 2 = 5, and SCC Grade 3 = 6) and then analyzed using a two-sided Wilcoxon rank-sum test. Statistical analysis was performed using MSTAT statistical software version 6.6.1 (https://oncology.wisc.edu/mstat/; last accessed 21 October 2020).

## 5. Conclusions

The causal relationship between MCPyV and MCC represents the first association of a human polyomavirus with human cancer. Understanding the role of this newly discovered DNA tumor virus and its viral proteins in the pathogenesis of MCC is critical to identifying prevention and therapeutic approaches to this aggressive and lethal cutaneous cancer. We have recently developed and characterized a MCPyV transgenic murine model that involves the targeted expression of MCC tumor-derived MCPyV-truncated LT and ST antigens to epithelial cells of murine skin [32]. In the study presented here, we utilized these transgenic mice to determine the role of the MCPyV T antigens in different stages of skin carcinogenesis. This well-validated, multi-stage model of skin cancer development uses topical application of chemical carcinogens to identify the role of genes and factors in tumor initiation, tumor promotion, and malignant progression. We found that the MCPyV T antigens function as tumor promoters, and not tumor initiators, in murine skin. The functions of the MCPyV T antigens in this in vivo assay closely mirrored the actions of viral oncoproteins from another DNA tumor virus, HPV16. It is possible that the MCPyV T antigens function differently in other potential MCC precursor cell types or when expressed under certain conditions within the milieu of human skin. However, in the context of epithelial tumorigenesis, these observations suggest that other factors likely contribute to tumor initiation and cooperate with the tumor-promoting functions of the MCPyV T antigens during MCC development.

## Figures and Tables

**Table 1:** Overview of treatment groups and number of mice per group at study onset and endpoint. Starting number of mice per each treatment group and genotype are shown. The number of mice remaining at 20 weeks post-DMBA treatment is shown in parentheses. Any change in the number of mice per group is accounted for in statistical tests at each time point.

Experimental Group	Treatment			
	TPA Only	DMBA Only	DMBA+TPA	No Treatment
*ROSA26-LSL-MCPyV168*	17	22 (18)	23 (19)	0
*K14Cre-MCPyV168*	12	14	17 (16)	28 (22)
*K14E6/E7*	13	10	22 (16)	0

**Table 2:** Histopathological scoring of disease in tumors arising on murine skin after treatment with chemical carcinogens. After completing 20 weeks of TPA treatment following one-time treatment with DMBA, mice were held for an additional 5 weeks to monitor malignant progression. The number of mice and total tumors/foci evaluated is indicated in parentheses. The number of foci scored as having each disease grade is indicated for each group of mice.

Disease Grade	Experimental Groups		
	*R26-LSL-MCPyV168* (n = 8 mice, n = 24 foci)	*K14E6/E7* (n = 6 mice, n = 20 foci)	*K14Cre-MCPyV168* (n = 7 mice, n = 26 foci)
Dysplasia Grade 1	12	6	14
Dysplasia Grade 2	4	6	4
Dysplasia Grade 3	0	2	2
SCC Grade 1	8	4	6
SCC Grade 2	0	1	0
SCC Grade 3	0	1	0

## Data Availability

The data presented in this study are available within this article.
